# CSF1R+ Macrophages and Osteoclasts Are Essential for Limb Bone Development During Embryogenesis

**DOI:** 10.3390/jdb14030031

**Published:** 2026-07-08

**Authors:** Felix Ma, Rose Ru Jing Zhou, Matthew Rosin, Jessica M. Rosin

**Affiliations:** 1 Craniofacial Science Graduate Program, Faculty of Dentistry, The University of British Columbia, Vancouver, BC V6T 1Z3, Canada; felixkhm@student.ubc.ca; 2 Life Sciences Institute, The University of British Columbia, Vancouver, BC V6T 1Z3, Canada; 3 Doctor of Dental Medicine Program, Faculty of Dentistry, The University of British Columbia, Vancouver, BC V6T 1Z3, Canada; 4 Department of Oral Biological and Medical Sciences, Faculty of Dentistry, The University of British Columbia, Vancouver, BC V6T 1Z3, Canada

**Keywords:** colony-stimulating factor-1 receptor (CSF1R), macrophage, osteoclast, limb morphogenesis

## Abstract

Colony-stimulating factor-1 receptor (CSF1R) provides essential signals for macrophage and osteoclast proliferation, differentiation, and survival, but the roles of CSF1R+ macrophages and osteoclasts during limb morphogenesis are understudied. Here, we utilized a pharmacological model by feeding the CSF1R inhibitor PLX5622 to pregnant mice across gestation to examine how CSF1R disruption impacts embryonic limb development. CSF1R-expressing cells were significantly depleted in response to PLX5622 exposure, including a complete loss of embryonic osteoclasts and osteoclastic activity in the developing limb bones. Although the gross morphology of limb nerves, muscles, cartilage, and bone appeared intact between embryonic day 11.5 (E11.5) and E15.5, prenatal PLX5622 exposure resulted in a completely penetrant truncated phenotype for all postnatal day 1 (P1) limb bones analyzed, suggesting that CSF1R+ cells play important roles in mediating limb bone formation during late embryogenesis. Interestingly, strain-specific defects were observed in the heel, where most of the CD1 mice presented with absent talus and underdeveloped calcaneus bones, while the C57BL/6 mice presented with milder developmental disruptions in both bones. Taken together, our data demonstrate that PLX5622 effectively depletes CSF1R-expressing macrophages and osteoclasts in embryonic limbs and suggest an essential role for embryonic CSF1R+ cells in driving limb bone morphogenesis.

## 1. Introduction

Colony-stimulating factor-1 receptor (CSF1R) is expressed in microglia, macrophages, and osteoclasts and is a crucial driver of their proliferation, differentiation, and survival [1,2,3,4,5,6,7,8,9]. CSF1R signalling is also required for the normal function of these cells, including macrophage phagocytosis and osteoclast bone resorption [6,10]. These essential signals provided by CSF1R have been studied in rodent models, where loss of CSF1R signalling leads to a hallmark osteopetrotic phenotype, which includes increased bone density, loss of bone marrow cavities, and subsequent loss of bone marrow hematopoiesis [11,12,13,14,15,16,17,18,19,20,21,22]. Specifically, mutations in colony-stimulating factor-1 (CSF1), the main ligand for CSF1R, have been identified in *Csf1 osteopetrotic* (*op*) mice and *Csf1 toothless* (*tl*) rats, resulting in identical phenotypes of toothlessness and delayed growth, leading to reduced body size and limb lengths [11,12,14,16,18,19,23,24,25,26,27,28,29]. Growth delays are also present in *Csf1r* KO mice and rats, leading to reduced body size and limb lengths [11,13,15,17,30,31,32,33]. Limb phenotypes have been noted in *Csf1r* KO rodents, with reduced limb bone mineralization observed in both mice and rats, especially in the secondary ossification centres [17,33]. In *Csf1r* KO mice, this reduced limb bone mineralization was characterized at postnatal day 14 (P14) [33], with placental and embryo development being reported as normal [34]. In *Csf1r* KO rats, there was also a complete loss of mineralization in the forelimb and hindlimb paws at birth, as well as loss of muscle mass by P7 due to reduced muscle fibre diameter [17]. Mutations in *Csf1* and *Csf1r* in both mice and rats cause significant reductions in tissue macrophages and loss of osteoclasts and osteoclastic tartrate-resistant acid phosphatase (TRAP) activity [11,12,13,14,15,16,17,18,19,24,27,28,34,35,36,37,38,39,40,41,42]. However, these genetic models exhibit compromised survival, as *Csf1* and *Csf1r* mutant mice present with reduced litter sizes and early postnatal lethality (up to 100% depending on the strain) [11,12,43]. Mutant rats show improved survival as all females survive beyond weaning, but 23% of males still die postnatally [13]. Importantly, while bi-allelic *CSF1R* mutations in humans result in brain abnormalities, they often also cause skeletal dysplasia within the dysosteosclerosis–Pyle disease spectrum [44,45,46]. Specifically, sclerosis of the pelvis and vertebrae is common, in addition to under-modelling and widening of the metaphyses and sclerosis of the diaphyses [45,46].

To examine how CSF1R+ cells contribute to embryonic development, we previously established a CSF1R pharmacological inhibition model using PLX5622, an orally bioavailable CSF1R inhibitor [47,48,49]. To disrupt CSF1R+ cells during embryogenesis, PLX5622-laced chow was fed to pregnant mice from embryonic day 3.5 (E3.5) to birth [47,48,49]. In response to PLX5622 exposure *in utero*, ~99% of hypothalamic microglia were depleted by E15.5 [49], and 21–80% of craniofacial macrophages were depleted by E15.5, depending on the structure examined [47], with an absence of embryonic osteoclasts and TRAP activity in E15.5 craniofacial bones [47]. This pharmacological mouse model demonstrates improved survival when compared with *Csf1* and *Csf1r* mutant mice, with only ~23% postnatal lethality observed [49]. Like the mutant mouse models, PLX5622 exposure also resulted in multiple craniofacial bone abnormalities [47,48,49]. Interestingly, strain-dependent responses to PLX5622 exposure were also described between CD1 and C57BL/6 mice, with milder craniofacial bone phenotypes observed in C57BL/6 mice [47]. However, previous PLX5622 studies have only focused on the brain and craniofacial region, and the depletion of CSF1R+ cells in the limbs was not characterized. As CSF1R disruption appears to strongly impact craniofacial bone development [47,48,49,50], and limb bone phenotypes have been observed but are understudied in mice, rats, and humans with *Csf1r* and *CSF1R* mutations [17,33,45,46], it is important to evaluate whether the PLX5622-exposed skeletal defects extend into the limbs. Particularly, CSF1R+ cell depletion in the embryonic limbs and its resulting impact on limb nerve, muscle, cartilage, and bone development across embryogenesis to birth have not been studied in rodent models. Further, PLX5622 allows for titratable CSF1R inhibition to limit postnatal lethality observed in mutant rodent models [11,12,43] and pinpoint phenotypes that are relevant to CSF1R signalling deficiency, which accounts for 8/11 human *CSF1R* mutations in BANDDOS [44], rather than the complete loss of signalling.

In this study, we investigated how prenatal PLX5622 exposure impacts nerve, muscle, cartilage, and bone development in the limbs across embryogenesis. Prenatal exposure to PLX5622 resulted in a significant depletion of CSF1R-expressing cells across gestation, with a complete loss of embryonic osteoclasts in the developing humerus and absence of TRAP activity in all embryonic limb bones. While we did not observe overt abnormalities in the morphogenesis of limb nerves, muscles, cartilages, and bones between E11.5 and E15.5, we identified a robust truncated phenotype in all limb bones at P1, suggesting that CSF1R+ cells play important roles in mineralization during late embryogenesis. We also observed strain-dependent responses to PLX5622 in the talus and calcaneus, in which the talus was absent and the calcaneus was underdeveloped in most of the PLX5622-exposed CD1 mice, but both bones were relatively mildly disrupted in the C57BL/6 mice. Together, these data show that CSF1R-expressing macrophages and osteoclasts in the embryonic limbs are significantly depleted in response to PLX5622 and suggest that these cells are crucial for normal limb bone morphogenesis.

## 2. Materials and Methods

### 2.1. Mouse Handling

The animal research received prior approval from the University of British Columbia’s Animal Care Committee (protocols A21-0170 and A21-0171) and was carried out in accordance with the guidelines and regulations of the Canadian Council of Animal Care. Mouse handling methods were adapted from Ma et al. [47]. In brief, CD1 (CR: 022; RRID: IMSR_CRL:022, Charles River) and C57BL/6 (JAX: 005304; RRID: IMSR_JAX:005304, The Jackson Laboratory) embryos and postnatal day 1 (P1) pups were utilized across this study, as described. Heterozygous *Csf1r^EGFP^* embryos were generated for flow cytometry and immunofluorescence experiments by crossing *Csf1r^EGFP^* mice (JAX: 005304; RRID: IMSR_JAX:005304, The Jackson Laboratory) with C57BL/6 mice. Embryonic samples were collected using timed pregnancies following detection of a vaginal plug, which was assigned embryonic day 0.5 (E0.5). Pregnant female mice were provided either a control diet (AIN-76A, Research Diets) or a diet containing the CSF1R inhibitor PLX5622 (1200 PPM added to chow AIN-76A, Research Diets) *ad libitum*, starting at E3.5 until sample collection. Pregnant dams were randomly assigned to the control or PLX5622 diet groups. To collect embryos, the pregnant female mice were anesthetized and immediately euthanized. For postnatal samples, P0 was assigned on the day of birth. Standard chow (LabDiet PicoLab Rodent Diet 20) was returned to the cages immediately following birth. Mouse pups were euthanized on P1. Two or more litters were collected for all embryo and pup analysis. Embryos/pups were randomly selected from collected specimens for all staining experiments. There were no exclusions for any experiments.

### 2.2. Genotyping

Genotyping methods were adapted from Ma et al. [47]. In brief, the tails were collected from all mouse embryos/pups for genotyping. The tail tissue was incubated in extraction buffer with proteinase K (New England Biolabs, Ipswich, MA, USA, Cat#P8107S; UniProtKB: P06873) overnight. Digested tail tissue was precipitated with saturated NaCl solution, centrifuged, the supernatant was collected, DNA was precipitated with isopropanol, and then DNA was pelleted by centrifugation. DNA pellets were washed, dried, and resolubilized in Tris-EDTA buffer. To amplify relevant sequences, DNA was added to OneTaq^®^ Quick-Load^®^ 2X Master Mix (New England Biolabs Cat#M0486S) containing primers for SX (*Sly* and *Xlr*) [51] or *GFP* [52] (see Table 1 for primer sequences). PCR reactions were run on an Applied Biosystems™ MiniAmp™ Thermal Cycler (Applied Biosystems, Waltham, MA, USA). PCR products were run on an agarose gel in TAE buffer and visualized by SmartGlow Pre-stain (Accuris Instruments Cat#E4500-PS) using a Fisherbrand™ Real Time Electrophoresis System (Fisher Scientific, Waltham, MA, USA). Amplified fragments were sized according to the GeneRuler Ready-to-Use 100 bp DNA Ladder (Thermo Fisher Scientific Cat#SM0243).

### 2.3. Flow Cytometry

Flow cytometry methods were adapted from Ma et al. [47]. In brief, mouse embryos at E11.5, 13.5, 15.5, and 17.5 were collected from the control and the PLX5622-exposed *Csf1r^EGFP^* mice in ice-cold PBS. Limb tissues were micro-dissected and transferred to ice-cold culture media containing DMEM (GIBCO 11965-092, Thermo Fisher Scientific), F-12 (GIBCO 11765-054, Thermo Fisher Scientific), and B-27 supplement (GIBCO 17504-044, Thermo Fisher Scientific). The limb tissue was dissociated, filtered at 35 μm, and centrifuged. The supernatant was discarded, and cell pellets were re-suspended in ice-cold HBSS/FBS. Immediately before analysis, cell suspensions were filtered at 35 μm. Flow cytometry analysis was carried out at the University of British Columbia’s Flow Core Facility using a Beckman Coulter Life Sciences CytoFLEX LX with Beckman Coulter CytExpert v2.4.0.28 software (Beckman Coulter Life Sciences [53]; RRID:SCR_017217).

### 2.4. Immunofluorescence Staining

Immunofluorescence staining methods were adapted from Ma et al. [47]. In brief, E15.5 *Csf1r^EGFP^* mouse embryos were collected in ice-cold PBS, and the limbs were micro-dissected. Following fixation in 4% paraformaldehyde (PFA) overnight, the limbs were washed, incubated in 30% sucrose/PBS overnight, then embedded in Clear Frozen Section Compound (VWR, 95057-838). The limbs were serially cryosectioned at 14 μm using a Leica CM1950 cryostat (Nussloch, Germany). The limb sections were washed, permeabilized, and blocked with donkey serum, then incubated with sheep anti-CSF1R (1:200; R&D Systems Cat#AF3818; RRID: AB_884158), rabbit anti-Cathepsin K (1:200; Abcam Cat#ab19027; RRID: AB_2261274), rabbit anti-Active Caspase 3 (1:500; BD Pharmingen Cat#559565; RRID: AB_397274), rabbit anti-Sp7 (1:500; Abcam Cat#ab209484; RRID: AB_2892207), rabbit anti-Collagen Type I (1:400; Proteintech Cat#14695-1-AP, RRID:AB_2082037), or mouse MF20 (1:100; Developmental Studies Hybridoma Bank Cat#MF20; RRID: AB_2147781) at 4°C overnight. The sections were washed then exposed to a secondary antibody (1:200; Alexa 594 donkey anti-sheep IgG (Invitrogen A11016), Alexa 594 donkey anti-rabbit IgG (Invitrogen A21207) or Alexa 594 donkey anti-mouse IgG (Invitrogen A21203)) for 2 h, washed, incubated with Hoechst 33342 (Invitrogen Cat#H3570; CAS: 23491-52-3) for 5 min, washed, and mounted with Aqua Poly/Mount (Polysciences Inc., Warrington, PA, USA). A ZEISS LSM900 confocal microscope was used to acquire fluorescent images, which were further processed using LSM+ deconvolution. The fluorescence intensity of MF20 immunostaining was quantified within a 100 µm × 100 µm square within the centre of the triceps and gastrocnemius muscles using ZEISS ZEN 3.9 (RRID:SCR_013672; ZEISS Microscopy [54]). If appropriate, the image brightness and/or contrast of the entire image was adjusted using ZEISS ZEN 3.9 (RRID:SCR_013672; ZEISS Microscopy [54]).

### 2.5. Whole-Mount Immunohistochemistry

Whole-mount staining methods were adapted from Ma et al. [47]. In brief, E11.5, 12.5, and 13.5 whole CD1 and C57BL/6 mouse embryos were collected in ice-cold PBS. The embryos were fixed overnight in Dent’s fixative at 4 °C, bleached overnight, serially rehydrated, blocked with skim milk proteins, and then exposed to antibodies recognizing either neurofilament (2H3; Developmental Studies Hybridoma Bank Cat#2H3; RRID: AB_531793) or muscle myosin (MF20) at 1:130–1:150 to achieve a final concentration of 2 μg/mL at 4 °C overnight. Embryos were then washed, blocked again, and then exposed to peroxidase-conjugated goat anti-mouse IgG (Sigma A-9169) at 1:300 at 4 °C overnight. The embryos were washed, stained with DAB (3,3′-Diaminobenzidine tetrahydrochloride; Sigma-Aldrich, St. Louis, MO, USA, Cat#D5905; CAS: 7411-49-6), dehydrated, and then cleared. A ZEISS Axiocam 208 colour camera (Jena, Germany) mounted on a ZEISS Stemi 508 microscope was used to capture images. The embryo limb lengths, limb nerve lengths, and limb muscle lengths and widths were measured using ZEISS Labscope 4.2 (RRID:SCR_021768; ZEISS Microscopy [55]). If appropriate, the image brightness and/or contrast of the entire image was adjusted using Adobe Photoshop CC v22.2.0.

### 2.6. Tartrate-Resistant Acid Phosphatase (TRAP) Staining

TRAP staining methods were adapted from Ma et al. [47]. In brief, E15.5 *Csf1r^EGFP^* mouse embryo limb cryosections (described above) were rehydrated, incubated in pre-warmed TRAP staining solution for 30 min at 37 °C, washed, and then mounted using Aqua Poly/Mount (Polysciences Inc.). A ZEISS Axiocam 208 colour camera mounted on a ZEISS Stemi 508 microscope was used to capture images. If appropriate, the image brightness and/or contrast of the entire image was adjusted using Adobe Photoshop CC.

### 2.7. Von Kossa Staining

Von Kossa staining methods were adapted from Ma et al. [47] and Tosun et al. [56]. In brief, E15.5 *Csf1r^EGFP^* mouse embryo limb cryosections (described above) were rehydrated, incubated in silver nitrate solution (Sigma-Aldrich Cat#209139; CAS: 7761-88-8) under direct light exposure for 1 h, washed, stained with Alcian blue solution (Sigma-Aldrich Cat#05500; CAS: 33864-99-2) for 20 min, stained with Nuclear Fast Red Solution for 10 min, washed, and then mounted with Aqua Poly/Mount (Polysciences Inc.). A ZEISS Axiocam 208 colour camera mounted on a ZEISS Stemi 508 microscope was used to capture images.

### 2.8. Bone and Cartilage Skeletal Staining

Skeletal staining methods were adapted from Ma et al. [47]. In brief, P1 CD1 and C57BL/6 mouse pups were collected and euthanized by decapitation. The pup carcasses were skinned, internal organs and fat tissue were removed, and then the carcasses were fixed in ethanol with 1% glacial acetic acid. The skeletons were incubated in Alcian blue solution (Sigma-Aldrich Cat#05500; CAS: 33864-99-2) overnight, washed, incubated in KOH solution for 3.5 h, incubated in Alizarin Red S solution (Sigma-Aldrich Cat#A5533; CAS: 130-22-3) for 3 h, cleared, and then stored and imaged in 40% glycerol. A ZEISS Axiocam 208 colour camera mounted on a ZEISS Stemi 508 microscope was used to capture images. If appropriate, the image brightness and/or contrast of the entire image was adjusted using Adobe Photoshop CC.

### 2.9. Quantification and Statistical Analysis

To obtain E15.5 limb tissue cryosections, PFA-fixed frozen embryo limbs were cryosectioned on a Leica CM1950 cryostat (Nussloch, Germany), as described above. Limb tissues were sectioned in the sagittal plane starting from the medial side of the limbs. Serial tissue cryosections were captured at 14 µm thickness across 10 slide sets, with 8 limb sections placed on each slide, until all limb tissue was collected. A single set of serial cryosections was used for each staining experiment described above. For *Csf1r^EGFP^*, CSF1R, cathepsin K, and cleaved caspase 3 cell counts, every 14 μm section containing the humerus was counted. Cells with positive expression were counted within the mineralizing ossification centre. A 20-cell radius peripheral to the entire cortical bone of the humerus was used for counting cells outside the ossification centre. TRAP+ area quantification methods were adapted from Ma et al. [47]. In brief, the images were adjusted to select for TRAP+ staining with Fiji v2.16 (RRID:SCR_002285; Schindelin et al. [57]) with ImageJ v1.54 (RRID:SCR_003070; Schneider et al. [58]) by using the *Color Threshold* function [57,58,59], converting to the *black and white (B&W)* mask, setting the *Image Type* to *8-bit*, selecting the TRAP+ stained area with the *Freehand selections* tool, eliminating outside pixels with the *Edit Clear Outside* function, and measuring the stained area using the *Analyze Particles* function. Quantitative data for all counts and measurements (*n* = 3–15 mouse embryos from 2–4 dams, unless otherwise mentioned in the Results and/or figure legends) were first assessed by two-way ANOVA where possible to identify any sex-dependent effects or interactions. As significant sex differences were minimal and did not show consistent changes (see Table 2 for significant sex-dependent effects and interactions), quantitative data for all counts and measurements were then pooled between sexes and colour-coded, with blue points representing males and pink points representing females. The data are represented by the mean ± SEM and were analyzed by a two-tailed unpaired *t*-test or the Mann–Whitney U test in GraphPad Prism 9 (RRID:SCR_002798; GraphPad Software [60]). Statistical significance was defined as *p* < 0.05.

**Table 2:** Significant sex-dependent effects and interactions in two-way ANOVA.

Figure	Description	Main Effect of Sex (*p*-Value)	Interaction Effect (*p*-Value)
Figure 1B, E11.5	Flow cytometry quantification of *Csf1r^EGFP+^* cells in E11.5 limbs	* *p* = 0.0156	*p* = 0.0793
Figure 1B, E13.5	Flow cytometry quantification of *Csf1r^EGFP+^* cells in E13.5 limbs	*p* = 0.3217	** *p* = 0.0050
Figure 2S	Quantification of TRAP staining in E15.5 ulna	* *p* = 0.0480	* *p* = 0.0480
Figure 3C	Quantification of E12.5 hindlimb lengths	* *p* = 0.0334	*p* = 0.4501
Figure 4N	Quantification of E12.5 ulnar nerve lengths	*p* = 0.1092	* *p* = 0.0437

All values *p* < 0.05 are bolded; * *p* < 0.05; ** *p* < 0.01.

**Figure 1:**
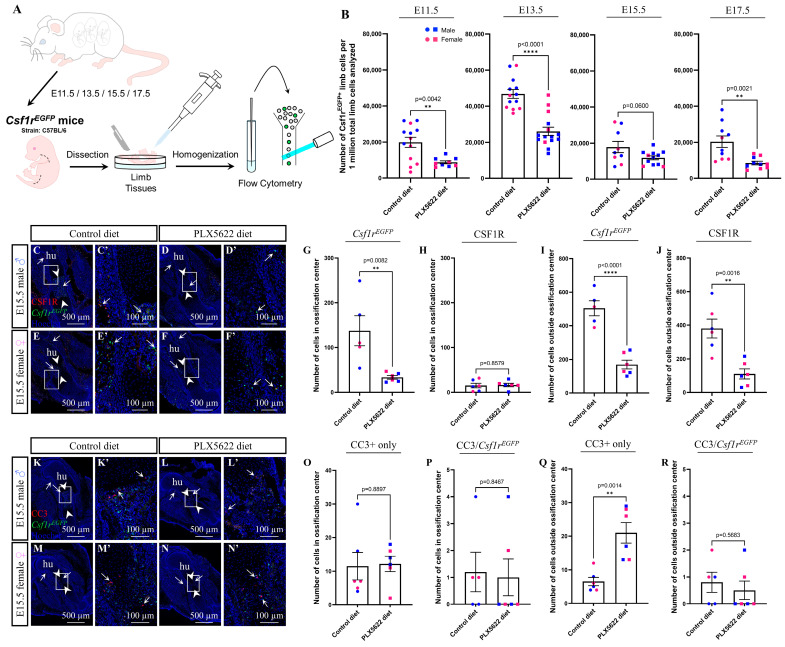
Prenatal exposure to PLX5622 significantly depletes CSF1R-expressing cells and increases apoptosis around the developing limbs. (**A**) A schematic illustrating the collection of *Csf1r^EGFP^*^+^ cells for flow cytometry. (**B**) Quantification of EGFP+ cells from *Csf1r^EGFP^* limb tissues (*n* = 3–10 embryos per sex/treatment/time-point from 2–4 dams). (**C**–**J**) Immunofluorescent images and quantification of *Csf1r^EGFP^+* and CSF1R+ cells within the mineralizing ossification centre (arrowheads) and around the E15.5 humerus (hu). The arrows mark *Csf1r^EGFP^* and CSF1R double-positive cells. (**K**–**R**) Immunofluorescent images and quantification of active cleaved Caspase 3+ (CC3+) and *Csf1r^EGFP^+* cells within the mineralizing ossification centre (arrowheads) and around the E15.5 humerus (*n* = 5–6 embryos per treatment from 2 dams). The arrows mark CC3 single-positive cells. The blue dots represent male embryos and the pink dots represent female embryos. Counts represent the mean ± SEM and were analyzed by a two-tailed unpaired *t*-test. ** *p* < 0.01; **** *p* < 0.0001.

**Figure 2:**
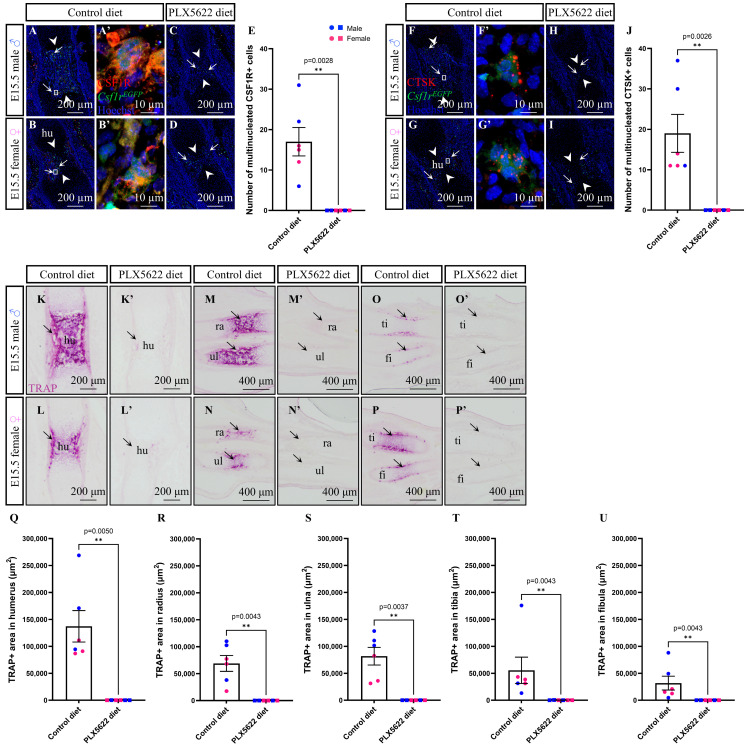
Prenatal exposure to PLX5622 disrupts osteoclast development and function. (**A**–**E**) Immunofluorescent images (**A**–**D**) and quantification (**E**) of multinucleated *Csf1r^EGFP^*/CSF1R double-positive osteoclasts (**A’**,**B’**) in the mineralizing ossification centre (arrowheads) of the E15.5 humerus. The arrows mark multinucleated *Csf1r^EGFP^*/CSF1R double-positive osteoclasts in the controls and the absence of osteoclasts in the PLX5622-exposed humerus bones. (**F**–**J**) Immunofluorescent images (**F**–**I**) and quantification (**J**) of multinucleated *Csf1r^EGFP^*/CTSK double-positive osteoclasts (**F’**,**G’**) in the mineralizing ossification centre (arrowheads) of the E15.5 humerus. The arrows mark multinucleated *Csf1r^EGFP^*/CTSK double-positive osteoclasts in the controls and the absence of osteoclasts in the PLX5622-exposed humerus bones. (**K**–**P’**) TRAP staining (arrows) in the E15.5 humerus (hu; **K**–**L’**), radius (ra), ulna (ul; **M**–**N’**), tibia (ti), and fibula (fi; **O**–**P’**). (**Q**–**U**) Quantification of the total TRAP+ area in the humerus (**Q**), radius (**R**), ulna (**S**), tibia (**T**), and fibula (**U**). *n* = 3 embryos per sex/treatment from 2 dams. The blue dots represent male embryos and the pink dots represent female embryos. Counts represent the mean ± SEM and were analyzed by the Mann–Whitney U test. ** *p* < 0.01.

**Figure 3:**
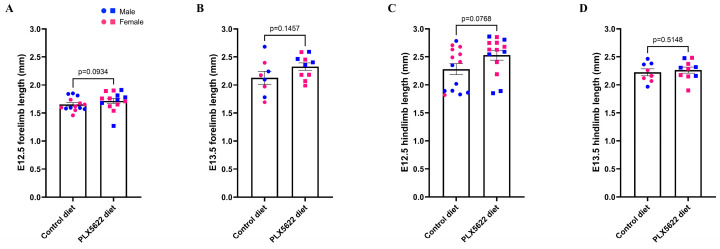
Prenatal exposure to PLX5622 does not affect limb lengths during embryogenesis. (**A**) Quantification of E12.5 embryo forelimb lengths (*n* = 6–8 embryos per sex/treatment from 2 dams). (**B**) Quantification of E13.5 embryo forelimb lengths (*n* = 4–6 embryos per sex/treatment from 2 dams). (**C**) Quantification of E12.5 embryo hindlimb lengths (*n* = 6–8 embryos per sex/treatment from 2 dams). (**D**) Quantification of E13.5 embryo hindlimb lengths (*n* = 4–6 embryos per sex/treatment from 2 dams). The blue dots represent male embryos and the pink dots represent female embryos. The measurements represent the mean ± SEM and were analyzed by the Mann–Whitney U test.

**Figure 4:**
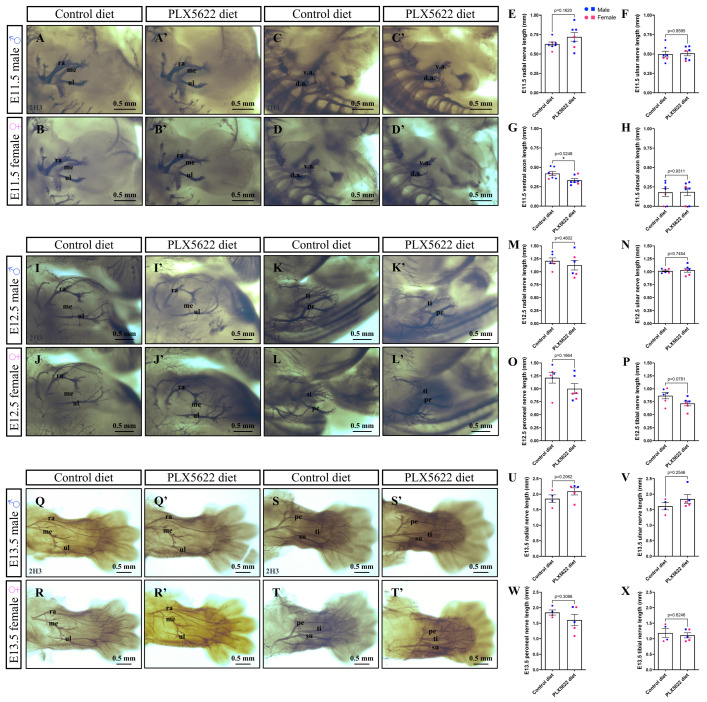
Prenatal exposure to PLX5622 does not impact nerve development. (**A**–**D’**) Whole-mount 2H3 immunostaining of E11.5 CD1 embryos. (**E**–**H**) Quantifications of E11.5 radial (**E**), ulnar (**F**), ventral axon (**G**), and dorsal axon (**H**) nerve lengths. (**I**–**L’**) Whole-mount 2H3 immunostaining of E12.5 CD1 embryos. (**M**–**P**) Quantifications of E12.5 radial (**M**), ulnar (**N**), peroneal (**O**), and tibial axon (**P**) nerve lengths. (**Q**–**T’**) Whole-mount 2H3 immunostaining of E13.5 CD1 embryos. (**U**–**X**) Quantifications of E13.5 radial (**U**), ulnar (**V**), peroneal (**W**), and tibial axon (**X**) nerve lengths. *n* = 4–7 embryos per treatment/time-point from 2 dams. The blue dots represent male embryos and the pink dots represent female embryos. The measurements represent the mean ± SEM and were analyzed by a two-tailed unpaired *t*-test. Abbreviations: d.a., dorsal axon; me, median nerve; pe, peroneal nerve; ra, radial nerve; su, sural nerve; ti, tibial nerve; ul, ulnar nerve; and v.a., ventral axon. * *p* < 0.05.

## 3. Results

### 3.1. Gestational Exposure to the CSF1R Inhibitor PLX5622 Depletes CSF1R+ Macrophages and Osteoclasts in Embryonic Limbs

As the CSF1R inhibitor PLX5622 was previously shown to effectively deplete macrophages and osteoclasts in embryonic craniofacial tissues [47], we sought to determine if similar depletion of these CSF1R-expressing cells could be observed in developing limbs. Accordingly, pregnant female mice were administered a control or PLX5622 diet starting at E3.5, as embryo implantation is dependent on endometrial macrophages [61,62], and continued up to embryo collection or birth. To quantify the proportion of CSF1R-expressing cells in the embryonic limbs, limb tissues were harvested from *Csf1r^EGFP^* transgenic male and female embryos, and cells with *Csf1r* promoter-driven enhanced green fluorescent protein (EGFP) signals were detected using flow cytometry (Figure 1A). *Csf1r^EGFP+^* cells were significantly depleted at E11.5 (Figure 1B; *p* = 0.0042) and E13.5 (Figure 1B; *p* < 0.0001) following PLX5622 exposure, with depletion levels ranging between 45% and 56%. Although there was a noticeable decrease in *Csf1r^EGFP+^* cells at E15.5 when comparing the control to PLX5622 male and female embryos (Figure 1B; *p* = 0.0600), this did not reach significance. In contrast, *Csf1r^EGFP+^* cells were again seen to be significantly depleted at E17.5 (Figure 1B; *p* = 0.0021), with only ~42% *Csf1r^EGFP+^* cells remaining in the PLX5622 male and female embryos.

To visualize CSF1R-expressing cell depletion in and around embryonic limb bones, we utilized both the *Csf1r^EGFP^* transgene and CSF1R immunofluorescence staining, as only ~92% of *Csf1r^EGFP+^* cells were previously found to also express CSF1R [47]. In the E15.5 *Csf1r^EGFP^* transgenic male and female limb cryosections that were stained with a CSF1R antibody, nuclear staining with Hoechst revealed a distinct primary ossification centre within the developing humerus (Figure 1C–F’; arrowheads). Within the ossification centre of the developing humerus, *Csf1r^EGFP+^* cells were significantly depleted by ~75% in response to PLX5622 exposure (Figure 1G; arrows; *p* = 0.0082). To our surprise, CSF1R+ cell numbers were comparable between the ossification centres of the E15.5 control and the PLX5622 male and female humeri (Figure 1H; *p* = 0.8579), as most *Csf1r^EGFP+^* cells in this region did not express the CSF1R protein. Outside of the ossification centre and surrounding the humerus, ~66% of *Csf1r^EGFP+^* cells (Figure 1I; arrows; *p* < 0.0001) and ~71% of CSF1R+ cells (Figure 1J; *p* = 0.0016) were significantly depleted following PLX5622 exposure.

Given that CSF1R+ cell depletion was previously shown to increase apoptosis in several embryonic craniofacial structures [47], and we observed a significant decrease in CSF1R-expressing cells in the embryonic limbs, we stained the E15.5 *Csf1r^EGFP^* transgenic male and female limb cryosections with an active cleaved Caspase 3 (CC3) antibody (Figure 1K–N’) to quantify changes in apoptosis in response to PLX5622 exposure. Within the primary ossification centre of the humerus, there were no significant differences in the number of CC3+ single-positive cells (Figure 1O; *p* = 0.8897) or CC3+/*Csf1r^EGFP+^* double-positive cells (Figure 1P; *p* = 0.8467). Intriguingly, there was a greater than three-fold increase in the number of CC3+ single-positive cells found outside the ossification centre (Figure 1Q; *p* = 0.0014); however, no change in the number of CC3+/*Csf1r^EGFP+^* double-positive cells was observed in this region (Figure 1R; *p* = 0.5683).

To quantify osteoclast-specific depletion, we examined multinucleated *Csf1r^EGFP^*/CSF1R double-positive cells within the primary ossification centre of the E15.5 male and female humeri (Figure 2A–B’; arrows) and found that PLX5622 exposure completely depleted these cells (Figure 2A–E; *p* = 0.0028). We further validated these findings by staining the E15.5 *Csf1r^EGFP^* transgenic male and female limb cryosections with a Cathepsin K (CTSK) antibody (Figure 2F–I; arrows). As expected, PLX5622 exposure resulted in complete depletion of *Csf1r^EGFP^*/CTSK double-positive multinucleated osteoclasts in the E15.5 male and female humeri (Figure 2F–J; arrows; *p* = 0.0026). Given the absence of CTSK+ osteoclasts in the limbs of the PLX5622 male and female embryos, we next examined osteoclastic tartrate-resistant acid phosphatase (TRAP) activity in the E15.5 *Csf1r^EGFP^* transgenic male and female limb cryosections. Consistent with our immunofluorescence staining, TRAP activity was absent in all of the E15.5 limb bones, including the humerus (Figure 2K–L’, 2Q; *p* = 0.0050), radius (Figure 2M–N’, 2R; *p* = 0.0043), ulna (Figure 2M–N’, 2S; *p* = 0.0037), tibia (Figure 2O–P’, 2T; *p* = 0.0043), and fibula (Figure 2O–P’, U; *p* = 0.0043). Taken together, these data demonstrate that gestational exposure to the CSF1R inhibitor PLX5622 significantly depletes CSF1R-expressing macrophages and osteoclasts in embryonic limbs, leading to a loss of osteoclastic TRAP activity across all embryonic limb bones and an accumulation of apoptotic cells surrounding the developing bony elements.

### 3.2. Prenatal Exposure to the CSF1R Inhibitor PLX5622 Does Not Impact Nerve or Muscle Development in the Limbs

As *Csf1r* knockout rats present with muscle fibre disruptions in the tibialis posterior postnatally as early as P7 [17], we were interested in investigating whether muscle development is impacted in the embryo by exposure to PLX5622 gestationally. We were also interested in examining nerve development, as soft tissues in the limbs are otherwise understudied in *Csf1*- and *Csf1r*-disrupted genetic models [11,12,13,14,26,43] and the PLX5622 pharmacological model [47,48,49,50,63]. Initial investigation of the male and female embryos exposed to PLX5622 demonstrated that they were not visually distinguishable from the control male and female embryos, with comparable forelimb lengths at E12.5 (Figure 3A; *p* = 0.0934) and E13.5 (Figure 3B; *p* = 0.1457), and hindlimb lengths at E12.5 (Figure 3C; *p* = 0.0768) and E13.5 (Figure 3D; *p* = 0.5148). Visualization of nerves in the limbs between E11.5 and E13.5 using whole-mount immunostaining and a Neurofilament antibody (2H3) displayed relatively comparable nerve development in the E11.5 male and female forelimb (Figure 4A–B’) and hindlimb (Figure 4C–D’) of the control and the PLX5622 CD1 embryos. Indeed, comparable radial (Figure 4E; *p* = 0.1620) and ulnar (Figure 4F; *p* = 0.8595) nerve lengths were observed in the forelimbs, while the ventral axon in the hindlimbs was found to be significantly shorter in the PLX5622 embryos (Figure 4G; *p* = 0.0248). Importantly, dorsal axon lengths were comparable between the control and the PLX5622 embryo hindlimbs (Figure 4H; *p* = 0.9311), demonstrating that not all hindlimb nerves were truncated in response to PLX5622 exposure. The development of forelimb (Figure 4I–J’) and hindlimb (Figure 4K–L’) nerves was also comparable between the E12.5 control and the PLX5622 male and female CD1 embryos. Indeed, no significant differences were observed between the control and the PLX5622 radial (Figure 4M; *p* = 0.4602) and ulnar (Figure 4N; *p* = 0.7454) nerve lengths in the forelimbs, and comparable peroneal (Figure 4O; *p* = 0.1664) and tibial (Figure 4P; *p* = 0.0781) nerve lengths were also seen in the control and the PLX5622 hindlimbs. Finally, comparable nerve development was observed between the E13.5 control and the PLX5622 male and female CD1 forelimbs (Figure 4Q–R’) and hindlimbs (Figure 4S–T’), with no significant differences found in nerve lengths of the radial (Figure 4U; *p* = 0.2062), ulnar (Figure 4V; *p* = 0.2546), peroneal (Figure 4W; *p* = 0.3086) and tibial (Figure 4X; *p* = 0.6246) nerves.

**Figure 5:**
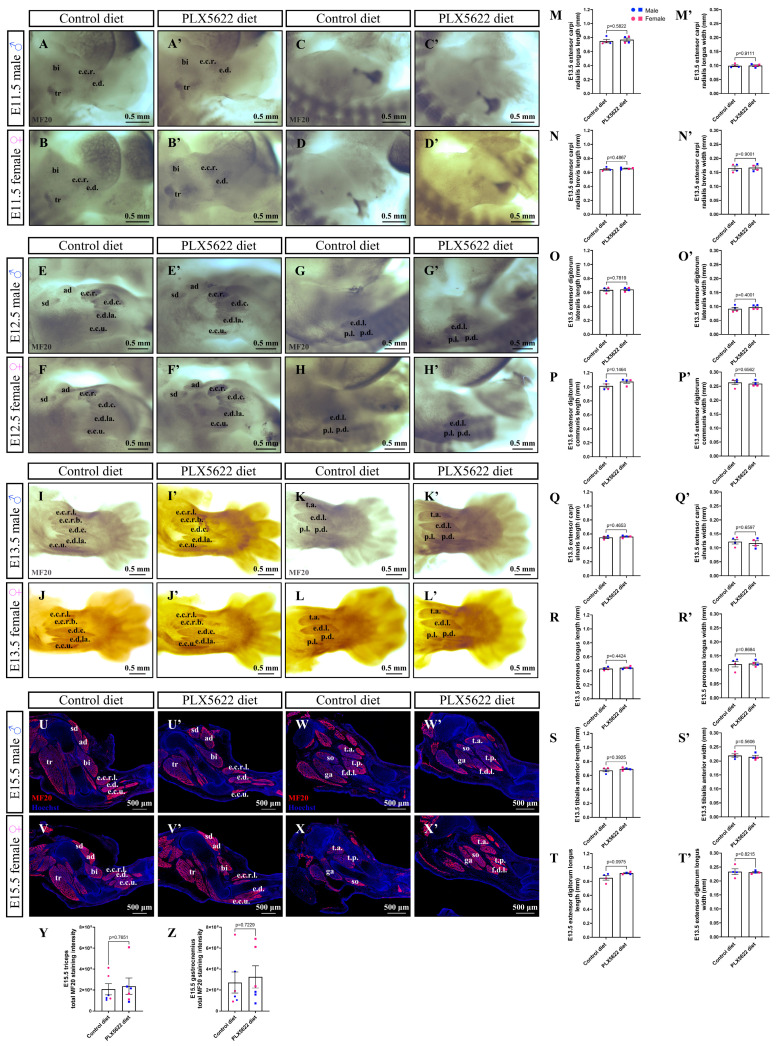
Prenatal exposure to PLX5622 does not impact muscle development. (**A**–**L’**) Whole-mount MF20 immunostaining of the E11.5 (**A**–**D’**), E12.5 (**E**–**H’**), and E13.5 (**I**–**L’**) CD1 embryos. Both forelimb (**A**–**B’**,**E**–**F’**,**I**–**J’**) and hindlimb (**C**–**D’**,**G**–**H’**,**K**–**L’**) muscle development appear comparable at each embryonic stage (*n* = 4–6 embryos per treatment/time-point from 2 dams). (**M**–**T’**) Quantifications of lengths and widths of the E13.5 extensor carpi radialis longus (**M**,**M’**), extensor carpi radialis brevis (**N**,**N’**), extensor digitorum lateralis (**O**,**O’**), extensor digitorum communis (**P**,**P’**), extensor carpi ulnaris (**Q**,**Q’**), peroneus longus (**R**,**R’**), tibialis anterior (**S**,**S’**), and extensor digitorum longus (**T**,**T’**) muscles. (**U**–**X’**) Immunofluorescent staining with MF20 of the E15.5 C57BL/6 limb tissue cryosections for the forelimbs (**U**–**V’**) and hindlimbs (**W**–**X’**). (**Y**,**Z**) Quantifications of MF20 immunofluorescence staining intensity in the E15.5 triceps (**Y**) and gastrocnemius (**Z**). *n* = 6 embryos per treatment from 2 dams. The blue dots represent male embryos and the pink dots represent female embryos. The measurements represent the mean ± SEM and were analyzed by a two-tailed unpaired *t*-test. Abbreviations: ad, acromiodeltoideus; bi, biceps; e.c.r., extensor carpi radialis; e.c.r.b., extensor carpi radialis brevis; e.c.r.l., extensor carpi radialis longus; e.c.u., extensor carpi ulnaris; e.d., extensor digitorum; e.d.c., extensor digitorum communis; e.d.l., extensor digitorum longus; e.d.la., extensor digitorum lateralis; f.d.l., flexor digitorum longus; ga, gastrocnemius; p.d., peroneus digiti; p.l., peroneus longus; sd, spinodeltoideus; so, soleus; t.a., tibialis anterior; t.p., tibialis posterior; and tr, triceps.

We then examined muscles in the limbs between E11.5 and E13.5 using whole-mount immunostaining and a Myosin Heavy Chain antibody (MF 20), observing comparable muscle development in the CD1 male and female embryos (Figure 5). Specifically, the biceps, triceps, extensor carpi radialis, and extensor digitorum were comparable between the E11.5 control and the PLX5622 male and female forelimbs (Figure 5A–B’). Overt muscle fibres could not be observed in the hindlimbs of the E11.5 male and female CD1 embryos (Figure 5C–D’). Visualization of muscles in the E12.5 limbs displayed comparable development of acromiodeltoideus, spinodeltoideus, extensor carpi radialis, extensor carpi ulnaris, extensor digitorum communis, and extensor digitorum lateralis muscles in the E12.5 male and female forelimbs (Figure 5E–F’) and extensor digitorum longus, peroneus longus, and peroneus digiti muscles in the the E12.5 male and female hindlimb (Figure 5G–H’) of the control and the PLX5622 CD1 embryos. Similarly, the extensor carpi radialis brevis, extensor carpi radialis longus, extensor carpi ulnaris, extensor digitorum communis, and extensor digitorum lateralis muscles were comparable between the E13.5 control and the PLX5622 male and female CD1 forelimbs (Figure 5I–J’), and extensor digitorum longus, peroneus longus, peroneus digiti, and tibialis anterior muscles were comparable between the E13.5 control and the PLX5622 male and female CD1 hindlimbs (Figure 5K–L’). Quantification revealed no significant differences in any of the E13.5 limb muscles, with comparable extensor carpi radialis longus lengths (Figure 5M; *p* = 0.5822) and widths (Figure 5M’; *p* = 0.9111), extensor carpi radialis brevis lengths (Figure 5N; *p* = 0.4867) and widths (Figure 5N’; *p* = 0.9001), extensor digitorum lateralis lengths (Figure 5O; *p* = 0.7819) and widths (Figure 5O’; *p* = 0.4001), extensor digitorum communis lengths (Figure 5P; *p* = 0.1464) and widths (Figure 5P’; *p* = 0.6562), extensor carpi ulnaris lengths (Figure 5Q; *p* = 0.4653) and widths (Figure 5Q’; *p* = 0.6597), peroneus longus lengths (Figure 5R; *p* = 0.4424) and widths (Figure 5R’; *p* = 0.8684), tibialis anterior lengths (Figure 5S; *p* = 0.3925) and widths (Figure 5S’; *p* = 0.5606), and extensor digitorum longus lengths (Figure 5T; *p* = 0.0975) and widths (Figure 5T’; *p* = 0.8215).

Next, to further investigate muscles deeper in the tissues and muscle fibre structure, we performed immunofluorescence staining on the E15.5 *Csf1r^EGFP^* transgenic male and female limb cryosections using an MF 20 antibody. Muscle fibre diameter and density appeared comparable in the acromiodeltoideus, spinodeltoideus, extensor carpi radialis longus, extensor carpi ulnaris, extensor digitorum, and triceps muscles in the E15.5 male and female forelimbs (Figure 5U–V’), as well as in the flexor digitorum longus, gastrocnemius, soleus, tibialis anterior, and tibialis posterior muscles in the E15.5 male and female hindlimbs (Figure 5W–X’). MF 20 immunofluorescent staining intensity within the E15.5 limb muscles was quantified, and no significant differences were found between the control and the PLX5622 triceps (Figure 5Y; *p* = 0.7651) and gastrocnemius (Figure 5Z; *p* = 0.7229). Collectively, the data suggest that prenatal exposure to PLX5622 during pregnancy does not disrupt nerve or muscle morphogenesis in the male or female limbs of CD1 or C57BL/6 embryos.

### 3.3. Embryonic Exposure to the CSF1R Inhibitor PLX5622 Disrupts Limb Bone Morphogenesis

**Figure 6:**
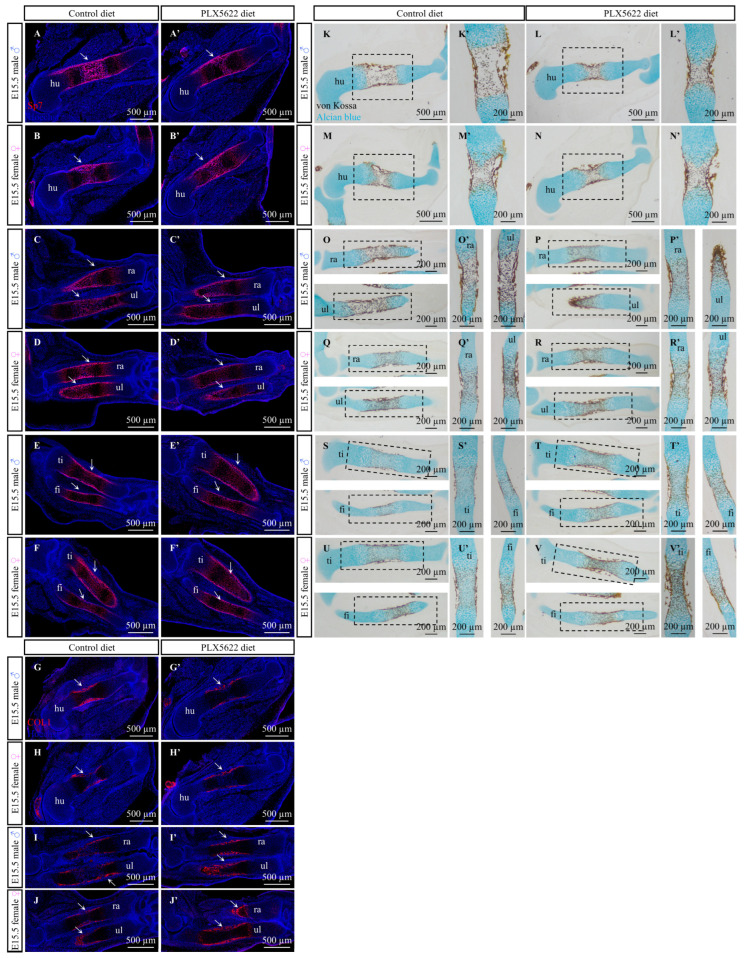
Prenatal exposure to PLX5622 does not drive overt bone phenotypes during embryogenesis. (**A**–**F’**) E15.5 Sp7 osteoblast staining (arrows) in the humerus (hu; **A**–**B’**), radius (ra), ulna (ul; **C**–**D’**), tibia (ti), and fibula (fi; **E**–**F’**). (**G**–**J’**) E15.5 type I collagen staining (arrows) in the humerus (**G**–**H’**), radius, and ulna (**I**–**J’**). (**K**–**V’**) E15.5 von Kossa and Alcian blue staining in the humerus (**K**–**N’**), radius, ulna (**O**–**R’**), tibia, and fibula (**S**–**V’**). *n* = 3 embryos per sex/treatment from 2 dams.

As prenatal exposure to the CSF1R inhibitor PLX5622 during pregnancy was previously shown to result in a number of craniofacial bone phenotypes in PLX5622 mice [47,48,49,50,63], with disruptions to embryonic osteoblast patterning and mineralization seen in several E15.5 craniofacial bones [47], we stained the E15.5 *Csf1r^EGFP^* transgenic male and female limb cryosections with an Sp7 antibody to visualize osteoblasts in the developing limbs. As expected, Sp7 osteoblast staining was highest around the primary ossification centre and the periosteum (Figure 6A–F’; arrows). However, unlike what was observed in craniofacial tissues [47], Sp7 staining appeared comparable in the humerus (Figure 6A–B’), radius, ulna (Figure 6C–D’), tibia, and fibula (Figure 6E–F’) of the E15.5 control and the PLX5622 male and female embryos. As Sp7 can also be expressed in osteoblast progenitors to induce type I collagen expression and form the extracellular matrix of bone [64,65,66], we also stained the E15.5 male and female limb cryosections with a type I collagen antibody to visualize whether osteoblast maturation was disrupted by PLX5622 exposure. Type I collagen staining appeared comparable in the humerus (Figure 6G–H’), radius, and ulna (Figure 6I–J’) of the E15.5 control and the PLX5622 male and female embryos, being mainly expressed in the periosteal bone collar surrounding the primary ossification centre, where Sp7 was also most highly expressed.

Next, we examined cartilage and bone development in embryonic limbs by performing Alcian blue and von Kossa staining on the E15.5 *Csf1r^EGFP^* transgenic male and female limb cryosections. Chondrocytes within the long bones could be visualized with Alcian blue staining, with larger hypertrophic chondrocytes observed adjacent to the mineralizing ossification centres (Figure 6K–V’); yet, overt differences in chondrocyte layers or cartilage patterning were not observed when comparing the humerus (Figure 6K–N’), radius, ulna (Figure 6O–R’), tibia, and fibula (Figure 6S–V’) of the E15.5 control and PLX5622 male and female embryos. As expected, von Kossa staining highlighted mineralization within the primary ossification centre and periosteal collar of each long bone in the developing limb (Figure 6K–V’). However, in contrast to what was observed in the craniofacial bones [47], no overt phenotypes were observed when comparing the humerus (Figure 6K–N’), radius, ulna (Figure 6O–R’), tibia, and fibula (Figure 6S–T’) of the E15.5 control and the PLX5622 male and female embryos.

**Figure 7:**
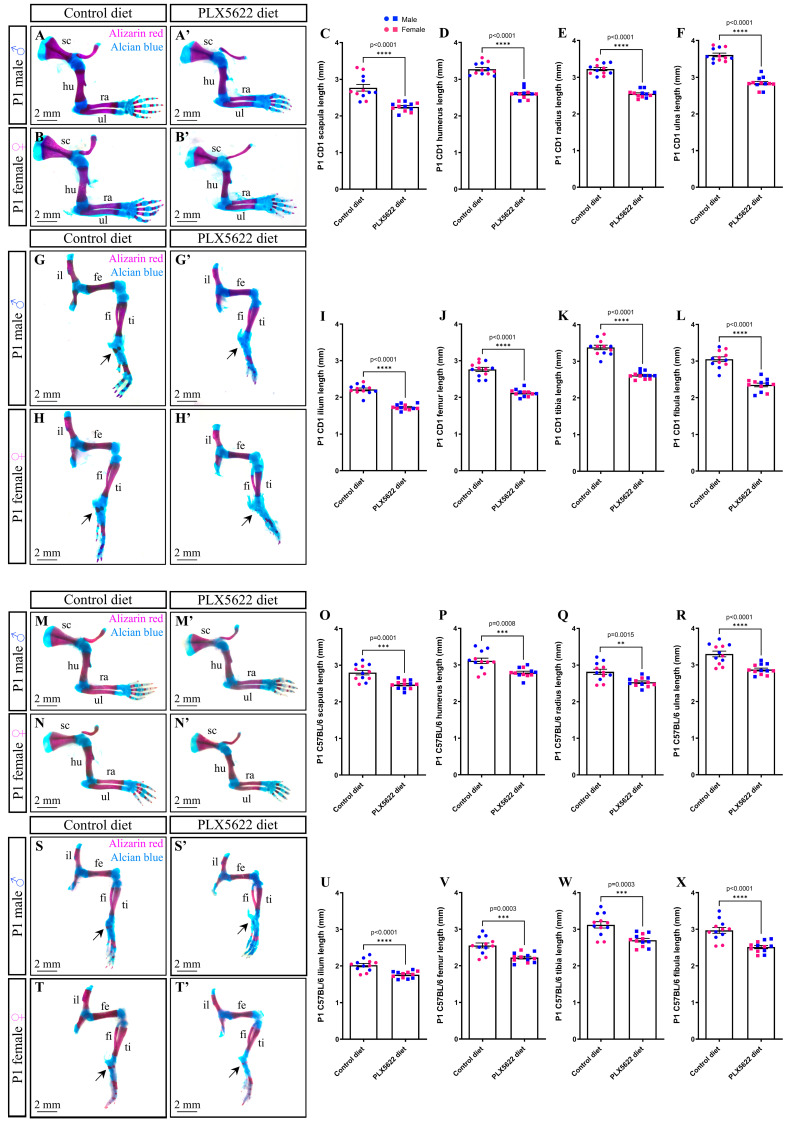
Prenatal exposure to PLX5622 disrupts limb bone morphogenesis. (**A**–**F**) A lateral view of the P1 CD1 forelimbs (**A**–**B’**) and quantification of the scapula (sc; **C**), humerus (hu; **D**), radius (ra; **E**), and ulna (ul; **F**) lengths. (**G**–**L**) A lateral view of the P1 CD1 hindlimbs (**G**–**H’**) and quantification of the ilium (il; **I**), femur (fe; **J**), tibia (ti; **K**), and fibula (fi; **L**) lengths. The arrows mark the talus and calcaneus bones in the heel, which are absent or underdeveloped in the CD1 PLX5622 pups. (**M**–**R**) A lateral view of the P1 C57BL/6 forelimbs (**M**–**N’**) and quantification of the scapula (sc; **O**), humerus (hu; **P**), radius (ra; **Q**), and ulna (ul; **R**) lengths. (**S**–**X**) A lateral view of P1 C57BL/6 hindlimbs (**S**–**T’**) and quantification of the ilium (il; **U**), femur (fe; **V**), tibia (ti; **W**), and fibula (fi; **X**) lengths. The arrows mark the talus and calcaneus bones in the heel, which are either absent or underdeveloped in the C57BL/6 PLX5622 pups. *n* = 6 pups per strain/sex/treatment from 3–4 dams. The blue dots represent male pups and the pink dots represent female pups. The measurements represent the mean ± SEM and were analyzed by a two-tailed unpaired *t*-test. ** *p* < 0.01; *** *p* < 0.001; **** *p* < 0.0001.

Considering that craniofacial bone defects could be observed postnatally in *Csf1*- and *Csf1r*-disrupted genetic models [11,12,13,14,26,43] and the PLX5622 pharmacological model [47,48,49,50,63], we sought to examine whether any limb bone phenotypes develop late in embryogenesis and could be observed in P1 CD1 and C57BL/6 limbs by staining with Alcian blue and Alizarin red to label cartilage and bone, respectively. Interestingly, all long bones of the limb were found to be shorter in the PLX5622-exposed pups (Figure 7). Indeed, in the P1 male and female CD1 forelimbs (Figure 7A–B’), scapulae (Figure 7C; *p* < 0.0001), humeri (Figure 7D; *p* < 0.0001), radii (Figure 7E; *p* < 0.0001), and ulnae (Figure 7F; *p* < 0.0001) were found to be significantly shorter in the PLX5622 pups as compared with the control pups. Similarly, in the P1 male and female CD1 hindlimbs (Figure 7G–H’), ilia (Figure 7I; *p* < 0.0001), femurs (Figure 7J; *p* < 0.0001), tibiae (Figure 7K; *p* < 0.0001), and fibulae (Figure 7L; *p* < 0.0001) were significantly shorter in the PLX5622-exposed pups. Notably, 9/12 of the P1 male and female CD1 PLX5622 pups also presented with absent talus and severely underdeveloped calcaneus bones in the heel, with the remaining 3/12 pups showing milder developmental disruptions (Figure 7G–H’; arrows). The C57BL/6 male and female forelimb bone lengths were also found to be significantly shorter in the PLX5622 pups (Figure 7M–N’), including the scapulae (Figure 7O; *p* = 0.0001), humeri (Figure 7P; *p* = 0.0008), radii (Figure 7Q; *p* = 0.0015), and ulnae (Figure 7R; *p* < 0.0001). Consistent with what was observed in the C57BL/6 forelimbs, the C57BL/6 male and female hindlimb bones (Figure 7S–T’), such as the ilia (Figure 7U; *p* < 0.0001), femurs (Figure 7V; *p* = 0.0003), tibiae (Figure 7W; *p* = 0.0003), and fibulae (Figure 7X; *p* < 0.0001), significantly decreased in length in response to PLX5622 exposure. In the C57BL/6 hindlimbs, the majority of the PLX5622 male and female pups also displayed underdeveloped talus and calcaneus bones, with only 2/12 of the pups having severely underdeveloped or absent talus and calcaneus bones (Figure 7S–T’; arrows). As a whole, these data suggest that while prenatal depletion of macrophages and osteoclasts using the CSF1R inhibitor PLX5622 does not appear to drive overt changes in osteoblast patterning and early bone formation by E15.5, consistent with the normal bone development reported in the E15.5 *Csf1r* KO mouse embryos [33], it does indeed disrupt limb bone development later during embryogenesis, thereby resulting in underdeveloped limb bones at birth, with comparable phenotypes to the reduced limb bone mineralization and truncated limbs observed later in *Csf1* and *Csf1r* mutant rodent models [11,13,14,15,16,17,18,19,33].

## 4. Discussion

This study is the first to characterize CSF1R+ macrophage and osteoclast depletion in limb tissues across embryogenesis, including the resulting limb phenotypes, in response to PLX5622 exposure [48,49,50,67,68,69,70,71]. CSF1R-expressing cells were significantly depleted in limb tissues across embryogenesis, where osteoclasts were particularly impacted by PLX5622 exposure, with a complete absence of embryonic osteoclasts seen in the developing humerus. Osteoclastic TRAP activity was also absent in all embryonic limb bones, including the humerus, radius, ulna, tibia, and fibula. Interestingly, CSF1R+ cell depletion did not appear to disrupt limb nerve, muscle, cartilage, or bone morphogenesis between E11.5 and E15.5. However, prenatal PLX5622 exposure caused a completely penetrant truncation of all limb bones visualized at P1, in addition to disruption of the talus and calcaneus in the heel, suggesting that CSF1R+ cell depletion disrupts osteogenic pathways during late embryogenesis. Although both the CD1 and C57BL/6 PLX5622 limbs were smaller and contained shorter bones compared with the control CD1 and C57BL/6 limbs, we also observed strain-dependent phenotypes, as the C57BL/6 PLX5622 limbs displayed milder disruption of the talus and calcaneus, which appeared absent or severely underdeveloped in most of the PLX5622-exposed CD1 pups. Taken together, these findings demonstrate that prenatal exposure to PLX5622 effectively depletes CSF1R-expressing macrophages and osteoclasts in developing limbs, which appear to play essential roles in limb bone morphogenesis.

Here, we employed a prenatal pharmacological mouse model of CSF1R disruption by feeding the CSF1R inhibitor PLX5622 to pregnant mice during gestation. Previous studies using this model have reported significant depletion of CSF1R-expressing cells across various embryonic tissues, including ~99% depletion of microglia in the embryonic hypothalamus and up to 80% depletion of CSF1R+ cells in and around developing craniofacial structures [47,49]. Here, we report 45–58% depletion of CSF1R-expressing cells across embryogenesis in whole limb tissue preparations analyzed using flow cytometry, with up to ~75% depletion of CSF1R-expressing cells seen in and around the developing E15.5 humerus, using immunofluorescence staining. To our surprise, we did not observe significant *Csf1r^EGFP+^* cell depletion in the E15.5 whole limb tissue preparations when quantifying using flow cytometry. This occurred following a rapid, approximately two-fold expansion of *Csf1r^EGFP+^* cells between E11.5 and E13.5, before returning to approximately initial levels by E15.5, suggesting that a wave of newly formed CSF1R-expressing cells could be more resistant to PLX5622. Indeed, erythro-myeloid progenitors (EMPs) migrate from the yolk sac and seed the fetal liver prior to E11.5, where they rapidly differentiate into erythrocytes, granulocytes, monocytes, and CSF1R+ macrophages during the second and final wave of embryonic hematopoiesis [7,72,73]. These EMPs remain in the fetal liver until E14.5 and give rise to new macrophages until E16.5 [72]. This correlates with the increase in *Csf1r^EGFP^*^+^ cells we observed between E11.5 and E13.5 and supports the idea that *Csf1r^EGFP+^* cells observed at E15.5 could contain more immature or newly differentiated macrophages that may be less susceptible to PLX5622. Nonetheless, these cells showed strong depletion levels by E17.5, either due to continued exposure to the CSF1R inhibitor or due to macrophage maturation of an earlier resistant population. The higher levels of depletion of CSF1R-expressing cells seen in and around the E15.5 humerus compared with what was observed in the whole E15.5 limb is also reminiscent of what was observed in and around bony and cartilaginous craniofacial structures [47], suggesting that CSF1R-expressing cells in and around bone and cartilage may be more susceptible to PLX5622 depletion than cells in soft tissues. The absence of embryonic osteoclasts in all of the PLX5622 limb bones analyzed highlights the susceptibility of bone-specific CSF1R-expressing cells to PLX5622 exposure.

Programmed cell death is a critical process during embryonic limb development, particularly in the apical ectodermal ridge and undifferentiated distal mesoderm of developing limb buds, to form the interdigital zones [74,75,76,77,78]. Macrophages phagocytose apoptotic cells and cellular debris [79,80], and macrophage clearance of apoptotic cell debris has been specifically studied in the embryonic limb buds [75]. Accordingly, the accumulation of CC3+ apoptotic cells surrounding the E15.5 PLX5622 humerus could be explained by the significant depletion of CSF1R+ macrophages in the PLX5622 limb tissues. The loss of such macrophage clearance and subsequent accumulation of apoptotic cells in the PLX5622-exposed embryos could result in perturbed developmental programs in the limb bud. Although we did not observe polydactyly or syndactyly in any of the PLX5622 pups, a decreased size of the phalanges was observed in both the male and female CD1 and C57BL/6 pups, just as all other limb bones were either reduced in size or absent.

The absence of osteoclasts observed in the limbs is a well-studied characteristic of CSF1R disruption, from PLX5622 pharmacological craniofacial studies to the various osteopetrotic *Csf1* ligand and *Csf1r* mutant models [11,12,13,14,15,16,18,19,27,36,37,38,39,40,41,42,47]. CSF1R mediates critical signals for osteoclast differentiation and function [11,13,81,82,83,84], so it is unsurprising that disruption of CSF1R signalling would inhibit osteoclastogenesis, resulting in a loss of osteoclasts and TRAP activity. Several other osteoclast-specific KO mouse models, such as *Tnfrsf11a* (RANK), *Tnfsf11* (RANKL), and *Acp5*, recapitulate the loss of osteoclast activity seen in our PLX5622 model and other genetic rodent models with disrupted CSF1R signalling [11,13,14,15,16,17,18,19] and result in truncated limb bones [85,86,87]. It is notable that milder CSF1R+ cell depletion observed in our prenatal PLX5622 exposure model still leads to a complete absence of osteoclasts, again highlighting what appears to be a greater susceptibility of bone-associated CSF1R-expressing cells or a higher threshold of CSF1R signalling required for osteoclastogenesis compared with macrophage differentiation. This loss of osteoclasts raises questions with respect to dysregulated bone remodelling signals important for recruiting osteoblasts [88,89,90,91,92]. Interestingly, embryonic osteoblast patterning appeared unimpacted by osteoclast absence in all of the E15.5 PLX5622 limb bones analyzed. Despite these findings, the PLX5622-exposed pups still displayed abnormal limb bones shortly after birth, suggesting that osteogenic pathways may be disrupted during late embryogenesis. Osteoclast-derived bone remodelling signals may not be necessary for chemotaxis of osteoblast progenitors to sites of limb bone formation during embryogenesis, but may yet be required for sustaining normal osteoblast function and mineralization. In fact, osteoclasts do express bone remodelling signals that influence maturation and mineralization in osteoblasts [33,93,94,95]. Similar to the present study, the absence of osteoclasts in genetic *Csf1* and *Csf1r* mutant rodent models also leads to truncated limb bones, increased limb bone density, decreased bone marrow volume, and reduced mineralization, observed particularly in secondary ossification centres of *Csf1r* KO mouse and rat limbs, described as early as P14 in mice and P7 in rats [11,13,14,15,16,17,18,19,20,21,22,33]. Interestingly, in the P1 PLX5622 skeletons, we did not observe a complete loss of mineralization in forelimb and hindlimb paws, which was observed in *Csf1r* KO rats [17]. In contrast, we only observed an absence of the talus bones in the hindlimb paws of most of the PLX5622-exposed CD1 mice, while all other bones were present but smaller in the PLX5622 forelimb and hindlimb paws. Perhaps the absence of osteoclasts in both the PLX5622 model and mutant rodent models results in truncated limb bones and reduced mineralization in the paws, but it is the extensive loss of other CSF1R-expressing populations (i.e., macrophages) in *Csf1* and *Csf1r* mutant rodents [11,13,15,16,17,19,24,28,34,35], either local or systemic, that disrupts crucial developmental signals to result in complete loss of mineralization.

In a prior study examining *Csf1r* KO rat limbs, decreased muscle fibre diameter was reported as early as 7 days to 7 weeks of age, resulting in decreased muscle mass [17]. Here, we observed comparable muscle development between E11.5 and E15.5. Although CSF1R-expressing macrophages have been demonstrated to be essential for muscle repair after injury in conditional CSF1R KO and CSF1R inhibitor mouse models [96,97], CSF1R+ macrophages also appear to mediate muscle damage and atrophy [96,98], suggesting that unique populations of macrophages could regulate muscle formation and degradation to maintain homeostasis. During embryogenesis, our findings suggest that CSF1R+ macrophage depletion does not disturb this homeostatic balance in limb muscles, thereby allowing for normal development. It is also possible that the decreased size and length of limb bones at birth alter mechanical forces during muscle contraction, which are critical signals for myogenesis from mesenchymal progenitors [99,100], later resulting in reduced muscle mass postnatally. A reduction in insulin-like growth factor-1 (IGF-1) signalling, which has been reported in *Csf1r* KO and *Csf1* toothless rats [13,17,40], could also drive the observed decreases in muscle mass, as IGF-1 is essential for muscle growth [101,102,103,104,105]. It is important to note, however, that prenatal exposure to PLX5622 did not significantly alter IGF-1 secretion or impact craniofacial muscle development embryonically [47], which could also be the case in embryonic limbs. If so, CSF1R+ cell depletion with PLX5622 during pregnancy may retain IGF-1 expression and allow for normal muscle development, or alternatively, the downregulation of IGF-1 may occur later in embryogenesis, and thus, muscle phenotypes are not observed between E11.5 and E15.5.

Despite the fact that studies in *Csf1* and *Csf1r* genetically-disrupted rodent models have yet to compare the impacts of genetic backgrounds on the resulting phenotypes [11,30,106], survival rates do indeed show an impact of strain: few inbred C57BL/6 survive to weaning, with slight survival improvement in mixed FVB/NJ, and 40–80% survival past weaning in C3H/B6/SvJ outbred strains [11,106]. Similarly, *Csf1r* KO inbred Dark Agouti rats show compromised survival compared with mixed or pure outbred Sprague–Dawley mutants [30]. Intriguingly, analysis of the impact of PLX5622 on craniofacial development in an outbred CD1 strain and an inbred C57BL/6 strain highlighted strain-dependent impacts, where milder craniofacial disruptions were observed in C57BL/6 mice when compared with CD1 mice [47]. In this study, where both CD1 and C57BL/6 strains were analyzed, we observed a milder reduction in heel bone mineralization of the talus and calcaneus in the C57BL/6 pups. Although inbred C57BL/6 mice appear to, in general, exhibit milder phenotypes in the PLX5622 pharmacological inhibitor model, they show higher rates of postnatal lethality in *Csf1* and *Csf1r* genetic mutant models, suggesting that there are likely strain-dependent differences in their development [107,108,109,110,111,112], which in turn respond uniquely to different mechanisms of CSF1R dysfunction.

Here, we showed that CSF1R-expressing cells are essential for normal limb bone development. Using a variety of staining methods, we showed that prenatal PLX5622 exposure does not appear to impact nerve, muscle, cartilage or bone development between E11.5 and E15.5, suggesting that the truncation in limb bones seen in P1 PLX5622 pups likely results from impaired osteogenic pathways during late embryogenesis. Taken together, our findings demonstrate that embryonic CSF1R+ macrophages and osteoclasts play important roles in directing normal bone morphogenesis in the limbs.

### Limitations of This Study

A potential limitation of our study is our use of the PLX5622 inhibitor to disrupt CSF1R signalling and deplete CSF1R-expressing cells. Of particular concern is possible off-target effects of the inhibitor on tyrosine kinases related to CSF1R, such as on FLT3, KIT, AURKC or KDR, which have been suggested to potentially mediate PLX5622-induced changes in brain endothelial cells [113]. However, these effects are only specific to the central nervous system [113]. Furthermore, cell-free enzyme assays have demonstrated that PLX5622 is more than 50-fold more effective against inhibiting CSF1R than KIT, AURKC or KDR, 20-fold more than FLT3 (no inhibition observed in cell-based assays), and did not significantly inhibit 196 other kinases tested [71]. Another recent study reported agonistic activation of constitutive androstane receptor (CAR) by PLX5622 [114]. PLX5622 was found to directly bind CAR with a dissociation constant of 2.52 μM [114]; however, PLX5622 potently inhibited CSF1R with an inhibitory constant of 5.9 nM [115], showing a much higher affinity for the correct target by orders of magnitude. Also, all *Csf1* and *Csf1r* genetic disruption rodent models have reported increased bone density [11,13,14,15,16,17,18,19], and increased bone density has also been observed in embryonic craniofacial bones following prenatal exposure to PLX5622 [47]. However, we were unable to characterize changes in embryonic bone density in limb tissue cryosections or clearly characterize postnatal bone density with skeletal staining. From our current P1 skeletal staining, the limb bones appear comparable in opacity between control and PLX5622-exposed pups; however, future studies should utilize micro-computed tomography technology to better characterize limb bone density.

## Figures and Tables

**Table 1:** Sequences of primers used for genotyping for sex and *Csf1r^EGFP^* transgene.

Primer Sequence	Source
SX (*Sly* and *Xlr*) forward primer: GATGATTTGAGTGGAAATGTGAGGTA	McFarlane et al. [51]
SX (*Sly* and *Xlr*) reverse primer: CTTATGTTTATAGGCATGCACCATGTA	McFarlane et al. [51]
*GFP* forward primer: AAGTTCATCTGCACCACCG	Tiscornia et al. [52]
*GFP* reverse primer: TCCTTGAAGAAGATGGTGCG	Tiscornia et al. [52]

## Data Availability

The original contributions presented in this study are included in the article. Further inquiries can be directed to the corresponding author.
